# Correction to: Emerging challenge: dynamic solution structures of nucleic acids

**DOI:** 10.1093/bfgp/elae053

**Published:** 2025-01-18

This is a correction to: Koichi Nishigaki, Emerging challenge: dynamic solution structures of nucleic acids, *Briefings in Functional Genomics*, Volume 18, Issue 3, May 2019, Pages 157–158, https://doi.org/10.1093/bfgp/elz015

In the originally published online version of the article, the incorrect authors were listed for this paper. The author list has now been emended in the online version to read: ``Koichi Nishigaki'' instead of: ``An-Liang Xia, Yong Xu, Xiao-Jie Lu''.

